# Assessment of Performance, Interpretability, and Explainability in Artificial Intelligence–Based Health Technologies: What Healthcare Stakeholders Need to Know

**DOI:** 10.1016/j.mcpdig.2023.02.004

**Published:** 2023-04-21

**Authors:** Line Farah, Juliette M. Murris, Isabelle Borget, Agathe Guilloux, Nicolas M. Martelli, Sandrine I.M. Katsahian

**Affiliations:** a Groupe de Recherche et d’accueil en Droit et Economie de la Santé Department, University Paris-Saclay, Orsay, France; b Innovation Center for Medical Devices, Délégation à la Recherche Clinique et à l’Innovation, Hôpital Foch, Suresnes, France; c Inserm, Centre de Recherche des Cordeliers, Sorbonne University, University Paris Cité, Paris, France; d Inria, Health data- and model- driven Knowledge Acquisition, PariSantéCampus, Paris, France; e Real World Evidence & Data Department, Pierre Fabre, Boulogne-Billancourt, France; f Department of Biostatistics and Epidemiology, Gustave Roussy, University Paris-Saclay, Villejuif, France; g Oncostat U1018, Inserm, University Paris-Saclay, Équipe Labellisée Ligue Contre le Cancer, Villejuif, France; h Hôpital Européen Georges Pompidou, Pharmacy Department, Paris, France; i Inserm, Centre d’Investigation Clinique 1418 (CIC1418) Épidémiologie Clinique, Paris, France; j Hôpital Européen Georges Pompidou (S.I.M.K), Department of Bioinformatics, Biostatistics and Public Health, Assistance Publique des Hôpitaux de Paris, Paris, France

## Abstract

This review aimed to specify different concepts that are essential to the development of medical devices (MDs) with artificial intelligence (AI) (AI-based MDs) and shed light on how algorithm performance, interpretability, and explainability are key assets. First, a literature review was performed to determine the key criteria needed for a health technology assessment of AI-based MDs in the existing guidelines. Then, we analyzed the existing assessment methodologies of the different criteria selected after the literature review. The scoping review revealed that health technology assessment agencies have highlighted different criteria, with 3 important ones to reinforce confidence in AI-based MDs: performance, interpretability, and explainability. We give recommendations on how and when to evaluate performance on the basis of the model structure and available data. In addition, should interpretability and explainability be difficult to define mathematically, we describe existing ways to support their evaluation. We also provide a decision support flowchart to identify the anticipated regulatory requirements for the development and assessment of AI-based MDs. The importance of explainability and interpretability techniques in health technology assessment agencies is increasing to hold stakeholders more accountable for the decisions made by AI-based MDs. The identification of 3 main assessment criteria for AI-based MDs according to health technology assessment guidelines led us to propose a set of tools and methods to help understand how and why machine learning algorithms work as well as their predictions.

**Article Highlights**

• The level of confidence in artificial intelligence (AI)-based medical devices relies on transparency (interpretability and explainability of outputs) and ethics (in terms of trustworthiness and regulation).
• To provide interpretability, we identified that metrics and methodologies for “explainable AI” need to be associated with ethical and legal analysis.
• Specific explainability and interpretability assessment by regulators increased and led to stakeholders being increasingly held accountable for the decisions made by AI-based medical devices.
• Acceptable standards for explainability are context-dependent and reliant on the risks in the clinical scenario.
• Raising awareness about these concepts is essential for their widespread adoption and to answer ethical questions.

Understanding of algorithms in general and in artificial intelligence (AI) in healthcare has become an essential criterion following the new regulation processes for AI (AI Act), data (General Data Protection Regulation), and medical devices (MDs) (Medical Device Regulation) in Europe. Among these, the AI Act is the first regulation to divide applications of AI into different risk categories: (1) unacceptable risk, (2) high risk, and (3) low or minimal risk.^1^

In medicine, AI can be used not only in combination with an MD but also as an MD by itself. In fact, MDs are defined in the European Medical Device Regulation as “any instrument, apparatus, appliance, software, implant, reagent, material, or other article intended by the manufacturer to be used, alone or in combination, for human beings for specific medical purposes.”^2^ Artificial intelligence–based MDs are health technologies employed to improve human capabilities for several applications, including prediction or identification of diseases, data classification or analysis for disease outbreaks, optimization of medical therapy, or disease diagnosis.^2^ The Food and Drug Administration (FDA) in the United States defines an AI-based MD as “Software as a Medical Device” when the algorithm is intended to prevent, diagnose, treat, mitigate, or cure diseases.^3^

An increase in approved AI-based MDs has been recorded, with 222 devices in the United States and 240 devices in Europe between 2015 and 2020.^4^ Methodological frameworks are designed by health technology assessment (HTA) agencies to assess these technologies, and these agencies aim to evaluate them using a standardized method through multiple domains, such as safety, clinical effectiveness, costs and economic evaluation, organizational aspects, patients, and social and legal aspects.^5^ The assessment of AI-based MDs is performed by health technology agencies, such as Haute autorité de santé (HAS) in France, the National Institute for Health and Care Excellence in the United Kingdom, or FDA in the United States. In addition to the usual technical, clinical, and health economics criteria used for MD assessment, the need for specific criteria to assess AI technologies in healthcare has been highlighted.^6^

For instance, in France, HAS has defined 42 criteria, classified into 4 categories, to assess AI-based MDs. The fourth category, on functional characteristics, includes, in addition to algorithm performance, the criteria of explainability and interpretability. In the United States, FDA takes into account either the real-world or the human–AI team performance, the latter of which relates to how interpretable the model outputs are for humans, with an emphasis on the performance of the model. The performance of AI technology is often prioritized; however, an inability to understand the algorithms raises serious concerns in terms of fairness, ethics, and trust, and both interpretability and explainability refer to this capacity to understand algorithms.

From a healthcare perspective, the opacity of some AI models led to a decline in adoption by healthcare professionals. Several authors have highlighted the need for making these AI-based MDs more interpretable; however, the authors have also insisted on the explainability for trustworthy AI.^7^, ^8^, ^9^ On the contrary, Ghassemi et al^10^ advocated the rigorous internal and external validation of AI models owing to the lack of suitable explainability methods. However, these notions seem to be important to develop trustworthy AI using several principles proposed by Hasani et al,^11^ such as transparency, explainability, technical robustness, or stakeholder involvement. Thus, there is a growing need for appropriate assessment methodologies for explainable and interpretable AI-based MDs.^12^

Therefore, the aim of this study was to specify the different concepts that are essential for the development of AI-based MDs and to shed light on how performance, interpretability, and explainability are key in the development of health technology models.

To meet this objective, we aimed to address these 3 fundamental aspects of the evaluation of all criteria involved in the development and use of such technologies. After presenting AI ecosystems in healthcare with a focus on HTA agencies (section 1: State of the art of the assessment of AI-based MDs by HTA agencies), we will examine how the performance of AI-based MDs is measured (section 2: How to measure the performance of AI-based MDs?) and then provide elements for integrating interpretability and explainability issues into the core of algorithm development (section 3: How can we evaluate interpretability and explainability in AI health technologies?). Finally, we will discuss the major relevance of these notions for all stakeholders and offer a decision-making tool to facilitate the HTA process (section 4: Discussion: to what extent can the explainability and interpretability of AI be as useful as performance for HTA?).

## State Of The Art Of The Assessment Of AI-Based MDs By HTA Agencies

The AI ecosystem involves a large diversity of stakeholders with heterogeneous competencies and knowledge essential to tackle the development, validation, assessment, and deployment of AI-based MDs. In addition to those who usually contribute to the creation of MDs and their assessment, the AI health sector includes new stakeholders specialized in data, information technology, and engineering: AI public research institutes (Supplemental Figure 1, available online at https://www.mcpdigitalhealth.org/). The AI healthcare area gathers multiple actors from different areas (health, information technology, robotics, the tech industry, and ethics). Therefore, a crucial step in assessing these technologies is to identify the various stakeholders and understand a common taxonomy and the key notions to bridge the gap between them, thereby guaranteeing a common basis for assessments. Assessing the requirements of different international HTA agencies related to the evaluation of AI-based MDs shows that, in addition to the usual HTA criteria, such as performance and safety, the need for interpretability is crucial for clinical diagnosis, prevention, or treatment. The need for explainability is also important to comply with the “right to explanation” provided by the European General Data Protection Regulation.

The European guidelines for trustworthy AI include the principles of explainability and interpretability in addition to fairness and prevention of harm.^13^

### Objective and Methods

A literature review, following Preferred Reporting Items for Systematic Reviews and Meta-Analyses recommendations, was performed to highlight the specific key criteria needed for an HTA of AI-based MDs (review protocol provided in Supplemental Material, available online at https://www.mcpdigitalhealth.org/).

### Results

Of 64 articles, 7 were selected after full-text screening. They included guidelines on HTA of AI-based MDs from 8 countries. For each guideline, the following 3 criteria were highlighted: performance, interpretability, and explainability (Table 1). Nevertheless, no methodology has been proposed to measure these criteria.

**TABLE 1:** Identification of Key Specific Criteria for Artificial Intelligence–Based Medical Device Assessment in the Reviewed Guidelines on Health Technology Assessment of Artificial Intelligence Technologies^a^

Country	Guidelines (date)	Criteria^b^	Description^b^	Reference, year^c^
**Finland**	**Digi-HTA: Health technology assessment framework for digital healthcare services (2019)**	**AI**	**Capacity of the staff to understand the operational logic of AI? (interpretability)** **Transparency of the conclusions and decisions of the AI solution, that is, understanding of medical staff about the origin of the decisions (explainability)**	**Haverinen et al, 2019**^78^
		Technical stability	The testing process and company’s process for handling error messages	
		Cost	Costs of using the product for a healthcare customer	
		**Effectiveness**	The product provides clinical benefits to the end users by improving their behavior related to their own health	
		Clinical safety	Risks, possible side effects, or other undesirable effects associated with using the product; research evidence available related to clinical safety	
		Data security	Information security and data protection requirements	
		Usability and accessibility	The process of the company to continue to evaluate and develop accessibility. Product compatibility with usability guidelines (if applicable)	
		Interoperability	The product interfaces into the website and software, the healthcare services, and electronic patient records	
		Robotics	Safety risks for healthcare personnel or customers and the robot’s design to avoid them	
**France**	**Liste des produits et prestations remboursables (LPPR) Guide: Dossier submission to the Medical Device and Health Technology Evaluation Committee (2020)**	Purpose	Specify the benefit of the information provided or decisions made by machine learning processes	**Haute autorité de sant, 2020**^81^
		Data	Describe samples used, input data involved for initial model learning or relearning, and input data involved in decision making	
		Model	Describe training, validation, and testing before and after MD deployment	
		**Functional characteristics**	**Performance** and qualification, system robustness and resilience, **explainability, and interpretability**	
**Australia**	**Clinician checklist for assessing suitability of machine learning applications in healthcare (2021)**	Purpose	Purpose of the algorithm	Scott et al,^63^ 2021
		Data	The quality of the data used to train the algorithm: accurate and free of bias, standardized and interoperable, and sufficient quantity of data	
		**Performance**	**Algorithm performance** **Algorithm transferability to new clinical settings** **Evidence generation related to the algorithm’s impact on patient care improvement and outcomes**	
		**Interpretability, explainability, and explicability**	**Clinically intelligible outputs of the algorithm: interpretability and explainability**	
		Workflow	Algorithm fitting into and complementing current workflows	
		Patient harm	Avoiding patient harm	
		Ethical, legal, and social	Ethical, legal, or social concerns raised by the algorithm	
**United States, Canada, United Kingdom**	**Good Machine Learning Practice for Medical Device Development: Guiding Principles**	**Product life cycle**	**Understanding of a model’s intended integration into clinical workflow (interpretability and explicability)** **Balance between desired benefits and associated patient risks** **Safety, effectiveness, and clinically meaningful needs addressed over the lifecycle of the device**	Korean Minis**US Food and Drug Administration, Health Canada, and the United Kingdom’s Medicines and Healthcare products Regulatory Agency, 2021**^82^
		Security practices	Good software engineering practices, data quality assurance, data management, and cybersecurity practices	
		Clinical study participants and datasets	Data collection: relevant characteristics of the intended patient population sufficiently represented in a sample of adequate size in the clinical study and training and test datasets, management of bias, promotion of appropriate and generalizable performance across the intended patient population	
		Training datasets/test sets	Training and test datasets were selected and maintained to be appropriately independent of one another	
		Selected reference datasets	Accepted, best available methods for developing a reference dataset, accepted reference datasets in model development and testing that promote and demonstrate model robustness and generalizability	
		Model design and intended use of the device	A model design supporting mitigation of known risks, such as overfitting, performance degradation, and security risks. Clinical benefits and risks are well understood, used to derive clinically meaningful performance goals for testing; the product can safely achieve its intended use	
		**Performance of the human–AI team**	Model as a “human in the loop,” consideration of human factors and the **human interpretability** of the model outputs are addressed with emphasis on the performance of the human–AI team	
		Device performance	Statistically sound test plans developed and executed to generate clinically relevant device performance information independently of the training dataset	
		**Clear and essential information for users**	Users are provided with ready access to clear, contextually relevant information that is appropriate for the intended audience (such as healthcare providers or patients), including the product’s intended use and indications for use, performance of the model for appropriate subgroups, user interface interpretation (**interpretability**), and clinical workflow integration of the model.	
		Performance and retraining risks	Capability to be monitored in “real-world” use with a focus on maintained or improved safety and performance	
**Europe (Greece)**	**Presenting AI, DL, and ML studies to clinicians and healthcare stakeholders: an introductory reference with a guideline and a Clinical AI Research checklist proposal (2021)**	Data	Outcome imbalances/training and testing/missing data/overfitting	**Olczak et al, 2021**^79^
		**Performance**	**Evaluation metrics**	
			**The confusion table**	
			**Measuring performance**	
			**Performance curves and AUC**	
			**Image segmentation or localization**	
			**Continuous measurements**	
			**Multiple measurements**	
		**Ethical considerations and methodological biases**	Data and privacy	
			Bias and fairness	
			Informed consent and autonomy	
			**Safety and interpretability**	
			Responsibility and liability	
**United States**		Study design	Clarity of the design	**Norgeot et al, 2020**^80^
	**Minimum information about clinical artificial intelligence modeling: the MI-CLAIM checklist (2020)**		Characteristics of the cohorts (training and test sets) and representativity of real-world clinical settings	
			Comparator	
		Data and optimization	Origin of the data, data quality, independence between training and test sets, data quantity, targeted population, input data type	
		**Model performance**	**Primary metric selected to evaluate algorithm performance (eg, AUC, F-score, etc)**	

			**Performance comparison between baseline and proposed model with the appropriate statistical significance**	
		**Model examination/assessment in clinical practice**	**Explainability: clinically intelligible outputs of the algorithm and explainability of the algorithm**	
			Algorithm fitting into and/or complementing current clinical workflows	
			Ethical, legal, or social concerns raised by the algorithm	
**South Korea**	**Guideline on Review and Approval of Artificial Intelligence (AI) and big data-based Medical Devices (For Industry)**	Characteristics	Medical device classification criteria	**Korean Ministry of Food and Drug Safety, 2020**^83^
		**Performance**	**Validate the essential requirements and clinical effectiveness**	
			**Clinical validation (clinical performance and efficacy)**	
		Cloud server	Technical specification: cloud server operating environment, cloud service type, security standard	
		Data	Output information, update cycle of training data and accuracy of diagnosis results in the main performance, data encryption and decryption, and policy on anonymity in the security specification	
		**Version control**	Management of product structure and design by a manufacturer and other management, such as addition of training data and **interpretability**	
		Management policy on learning data	Policy on data management to maintain the effectiveness of training data consistently and the timing for updating training data/data management organizations is required to set the quality control items and scope and criteria related to training data, and assess the quality of product algorithm	

a AI, artificial intelligence; AUC, area under the curve; DP, deep learning; HTA, health technology assessment; MD, medical device; ML, machine learning.

b The lines highlighted in bold correspond to the specific criteria related to artificial intelligence–based medical devices in each guideline.

c Our review selected 7 articles (out of 64), including guidelines on health technology assessment of artificial intelligence–based medical devices from 8 countries. For each guideline, 3 criteria are highlighted in green: performance, interpretability, and explainability.

On the one hand, some HTA agencies only focus on interpretability. On the other hand, other agencies, such as HAS in France, highlight these notions as essential to be defined in the reimbursement dossiers of AI-based MDs that are submitted by companies. Interpretability is an important criterion; assessors ask for the parameters that influence the decision and for the methods used to identify them. For explainability, this agency focuses on understanding the factors that lead to the decision-making process.

Even when there is no legal obligation, it is important for HTA agencies and clinicians to be able to justify their decision-making process to patients.^14^, ^15^, ^16^ Explainability allows comparisons of algorithms with current recommendations; however, explaining how the predictions are derived can be a time-consuming process and, hence, could be suggested in specific situations. For AI-based MDs with high risks for patient safety (for instance, those that impact morbimortality), explanations are vital. In addition, explanations can be required when an algorithm’s clinical performance has not yet been proven.^17^

Therefore, the next part of this study focused on the methods and tools used to assess performance, interpretability, and explainability to answer the need in an HTA process.

## How To Measure The Performance Of AI-Based MDs?

In this section, we outline which tools are available for measuring the performance of an algorithm and how to use them.

### Definition

Performance measurement consists of evaluating the error of the model (hence the reliability) by assessing the difference between predicted and observed data. It is usually based on a score, an error metric for which lower values indicate better results.

### Tools for Measurement

Various metrics exist to evaluate the performance of a model. Each meets different purposes according to the global objective of the modeling strategy (regression or classification).^18^ The list of metrics presented in the Supplemental Table (available online at https://www.mcpdigitalhealth.org/) is not exhaustive because the number of metrics is currently exploding to meet the needs of new applications. Some metrics are based on mean differences between estimated and true values (such as mean square-errors and *R*^*2*^-like measures; this is called calibration. Besides, discrimination describes the capacity of algorithm estimates to distinguish between individual observations, which does not imply to know whether the output is true.^19^ In any case, all metrics are subject to some limitations that should be outlined in the development of AI-based MDs (for further literature, see the online book from Biecek and Burzykowski^20^).

### Evaluation

The goal is to learn an algorithm that best maps input data to the outcome. The learning process consists of 3 main components: the space of assumptions, training data, and the loss function. The space of assumptions describes the overall authorized set for the algorithm. The training data include the set of input data and outcome used by the learning algorithm to adjust for the best parameters. The loss function measures the error between true and predicted outcomes. The relationship between the complexity of the space of assumptions, the size of the training data and the generalization error of the learned algorithm defines the bias-variance trade-off, which is both a fundamental concept and a key challenge. The generalization error is the difference between the expected error of the learned function on new data and the training error on the data used to learn the function. We assume in this section that training and test data are independent and identically distributed.

#### The Bias-Variance Trade-off

It is generally accepted that evaluating the algorithm on the same data it has learned on is a methodological mistake.^21^^,^^22^ Overfitting is when a model is able to predict perfectly well on fitted data but not on yet unseen data. When a model overfits, it typically leads to higher prediction errors because the model is too specific for the data and is barely generalizable. Predictions for individuals already in the database will rationally match with themselves, and therefore, there will be no prediction error. However, should there be small fluctuations in the training data, some error would be introduced by the sensitivity of the algorithm. This is called variance, which is highly dependent on small variations within the training sample. High variance with great capacity in fitting training data leads to overfit, whereas small variance has a small capacity to fit the training data and will underfit.

The opposite problem is bias, when error is introduced by approximating a complex problem using a simpler algorithm. High bias has a small capacity to fit the training data and will underfit, whereas low bias with a great capacity in fitting training data leads to overfit. The bias-variance trade-off is the balance between these 2 sources of error. A good trade-off point is achieved when the algorithm has low bias and low variance, which corresponds to a good balance between fitting the training data and generalizing to new data.

We have listed some best practices around the bias-variance trade-off and summarized them in Supplemental Figure 2 (available online at https://www.mcpdigitalhealth.org/).

#### Which Data to Use, When, and How

A common practice to avoid overfitting is to evaluate the algorithm on a random sample held outside of the data used to train it.^23^ The main idea is that the data on which the predictive model is applied, known as the test data, should be different from the training data. A systematic way to evaluate the aforementioned trade-off is an iterative split called cross-validation, in which the dataset is divided into different subsets and the model’s error is measured on each subset.^24^

Settings of the algorithm are commonly called hyperparameters and drive the inherent complexity in controlling the learning process.^25^ Hyperparameter optimization hence allows to find the optimal complexity of the algorithm that performs best both on the training and unseen data. The idea is to find the hyperparameter combinations that optimize the cross-validation metric. More inputs on hyperparameter optimization are given in the Supplemental Material (available online at https://www.mcpdigitalhealth.org/).

The final evaluation can be performed either on a test set previously held out or on external data.

## How Can We Evaluate Interpretability And Explainability In AI Health Technologies?

The following methods are intended to provide an understanding of model prediction and behavior as part of an evaluation dossier. They do not cover how the methods can be used to debug or improve a model. Therefore, interpretability and explainability are ideals to be achieved, rather than assets.

### Definitions

Artificial intelligence raises numerous questions because of its opaque decision-making process. Both interpretability and explainability aim to help understand algorithms and answer user-based questions regarding AI’s input, output, and performance (such as why, how, what if, and why not).^26^

Existing definitions for explainability and interpretability have been previously and widely discussed in the literature, and it seems that there is no clear taxonomy of concepts.^17^^,^^26^, ^27^, ^28^ Even though some authors consider the 2 concepts to be similar, some HTA agencies distinguish between them during the evaluation process (Table 1). Following are the definitions proposed by Markus et al^17^ in 2021:

1. “An AI system is explainable if the task model is intrinsically interpretable or if the non-interpretable task model is complemented with an interpretable and faithful explanation.”
2. “An explanation is interpretable if the explanation is unambiguous, i.e., it provides a single rationale that is similar for similar instances, and if the explanation is not too complex, i.e., it is presented in a compact form.”

If a task model is interpretable, it is hence very likely to be explainable.

### Tools for Measurement

**FIGURE 1:**
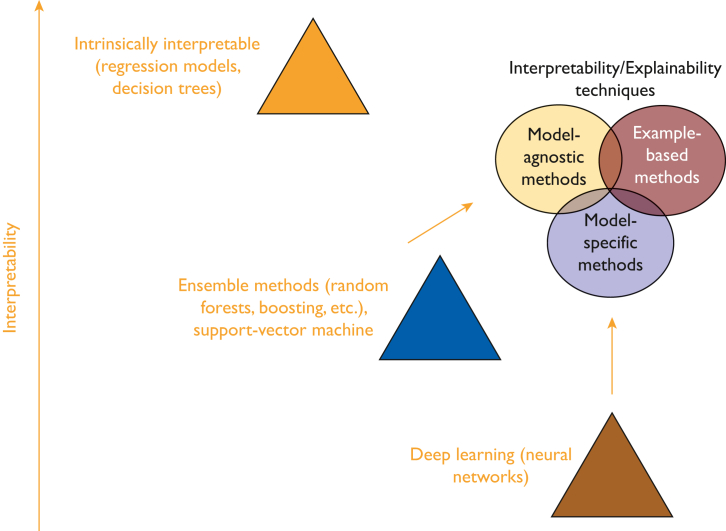
Tools to increase the interpretability and explainability of some well-known models.

Interpretability is difficult to define mathematically. Although there are many different machine learning (ML) algorithms, not all of them are explainable straightforwardly. However, 3 levels of interpretability (high, medium, and low ) have been identified. We extended the figure proposed by Dam et al^29^ (2018) by including existing tools that can increase the interpretability of the most well-known models while taking into account that black-box algorithms do not necessarily lead to higher performance (Figure 1).^30^ Figure 1 was not generated from any real data, and the y-axis has no quantification.

#### Interpretable by Design

Some models are interpretable by design under specific constraints, such as monotonicity, causality, and additivity (Table 2).^31^ They indeed already include internal functioning ready for interpretation, that is, they are intrinsically interpretable. Table 2 also provides relevant examples of application in the healthcare sector. Regression models are tangible equations with interpretable coefficients that can be read as linear coefficients with linear models, odds ratios for logistic regressions, and hazard ratios in Cox models to handle time-to-event data.^32^ Decision trees include interpretable rules and are greatly adapted to human thinking.^33^^,^^34^ Such methods should be the accepted baseline owing to their simple and fast processing and are highly preferable to any extremely complex model.^30^

**TABLE 2:** Models with a High Interpretability Level

Algorithm	Linear explanation	Monotone relationship	Task	Interpretable coefficient	Examples in healthcare (specialty, pathology, intended use of algorithm)
Linear regression	Yes	Yes	Linear	Linear coefficient	• Endocrinology, diabetes, prediction of severity (Butt et al, 2021)^84^ • Genetics, prediction of gene expression (Zeng et al, 2017)^85^
Logistic regression	No	Yes	Classification	Odds ratio	• Gastroenterology, fatty liver, prediction of disease in the general population (Bedogni et al, 2006)^86^ • Radiology, breast cancer, computer-aided diagnostic system (Nemat et al, 2018)^87^
Cox regression model	Yes	Yes	Time to event	Hazard ratios	• Cardiology, heart failure, prediction of mortality (Cheng et al, 2017)^88^ • Oncology, gastric cancer, prognosis prediction (Wei et al, 2021)^89^
Decision trees	No	Yes	All	Nodes	• Psychiatry, mental disorders, risk prediction (Van Hoffen et al, 2020)^90^ • Cardiology, malignant ventricular arrhythmia, diagnosis prediction (Mandala et al, 2020)^91^

#### Post Hoc Explanations

Ensemble methods (such as random forests or boosting), support-vector machines, or deep neural networks are uninterpretable algorithms. Post hoc explanations can either be global or local. Global explanations relate to the algorithm’s overall behavior, typically considering the overall importance of the covariates or features, and provide insight into how the algorithm makes predictions on a general basis. Conversely, local explanations refer to explanations at the scale of specific data points, detailing the reasons why the model chose these particular outcomes. Many toolkits and classifications are available in the literature to better describe how such post hoc explanations work.^35^, ^36^, ^37^, ^38^

In this section, we decided to list only the most well-known approaches of post hoc explanation. Because there is a growing need for interpretability to manage the exponential growth of the number of parameters in models, many approaches have been developed recently, and several typologies exist to classify them.^39^, ^40^, ^41^ Model-specific methods will not be discussed here because they depend highly on the model used for the prediction (such as gradient-based saliency maps, which are typically used for neural networks and imagery and providing each pixel’s importance). The main advantage of model-agnostic methods is that they can be applied in a post hoc manner to any kind of ML model.

Advantages and disadvantages for each method as well as relevant examples in healthcare are provided in Table 3. Overall, any element that can help understand the choices made by the AI algorithm are very welcome (eg, the study by Selvaraju et al^42^). Methods are yet to be made readily accessible to all stakeholders, from the developer to the end user. Work is currently underway to address this matter.^43^, ^44^, ^45^, ^46^

**TABLE 3:** Pros and Cons of Methods Serving Interpretability and Explainability^a^

	Easy to understand		Computation time	Data type	Limitations	Examples in healthcare (specialty, pathology, intended use of algorithm)
	For engineers^b^	For end users^c^				
Feature importance, SHAP, LIME	Yes	Intermediate, rarely shown	Low	Image, text, or tabular	Feature importance—sensitive to multicollinearity SHAP—sensitive to categorical variables and feature interactions LIME—difficulty in setting distance threshold	• Cardiology, cardiac surgery–associated acute kidney injury, prediction (Tseng et al, 2020)^92^ • Computational neuroscience, brain age prediction (Lombardi et al, 2021)^93^ • Pediatric medicine, organ transplantation, prediction of posttransplant health outcomes (Killian et al, 2011)^94^
Counterfactual explanations	Yes	Yes	High	Image, text, and mainly tabular	Difficulty in generating feasible and actionable explanations. Causal constraints	• Neurology, prediction errors in the human brain (Boorman et al, 2011)^95^

a LIME, Local Interpretable Model-agnostic Explanations; SHAP = Shapley Additive exPlanations.

b The engineers include, but are not limited to, developers, data scientists, and statisticians.

c The end users include, but are not limited to, healthcare professionals, decision-makers, medical experts, and patients.

### Evaluation of Performance, Interpretability, and Explainability

According to Ossa et al,^47^ in some cases, fewer explanations are acceptable if the risk-to-benefit ratio is clearly defined. Low-stakes decisions can tolerate less explainable AI as long as the mortality and morbidity risks are limited. In contrast, the diagnosis of a fatal disease requires that the AI algorithm provide doctors and patients with a complete understanding of its decision. The conceptualization of explainability in healthcare seems to be driven by and should focus on the context of clinical implementation. To date, no consensual approach exists for the evaluation of interpretability. However, Doshi-Velez and Kim^48^ have performed rigorous evaluation of interpretability and explainability, and their findings are outlined in the following sections.

#### Evaluations Involving Humans

First, application-grounded evaluation ensures that the algorithm performs the task for which it is designed by conducting human experiments. The principle is to involve end users (eg, physicians or radiologists) and show them explanations provided by the algorithm. The second step is to ask them what the machine would do and then present them with the actual output of the machine, working through a real-world example. By giving such tasks, you can quantitatively assess the difference in the performances of the humans and the model. Including both outliers and false assumptions in the algorithm also helps in spotting the expected outcomes. This constitutes a straightforward way of validating the objective, and, hence, the success of the algorithm’s performance. Application-level evaluations are yet to be deployed in healthcare.^49^, ^50^, ^51^

Second, human-grounded evaluation is similar to application-grounded evaluation but provides a simpler framework. The people involved are not experts anymore but lay people. Such experiments are typically recommended when objectives are wider than the assessment of interpretability/explainability of an algorithm. They are also cheaper because they do not require the involvement of high-level experts.

#### Evaluations Not Involving Humans

Functionally grounded evaluation does not involve human intervention. The aim is to formalize the algorithm’s components as an indicator of the quality of the explanation, favoring ease of use and simplicity. For example, a tree with a small depth is preferable to another with a large depth. Easy to formalize, function-based evaluation helps and is a valuable addition to human-based strategies.

Numerous measures to evaluate interpretability and explainability are emerging in the literature, including stability, simplicity, and faithfulness.^41^^,^^52^, ^53^, ^54^, ^55^, ^56^ Further guidance is also available elsewhere in the literature.^17^^,^^39^ Notably, the authors agree on the impossibility of fulfilling all properties for “good” explanations. However, human-based experiments are highly recommended whenever possible.

## Discussion: To What Extent Can The Explainability And Interpretability Of AI Be As Useful As Performance For Hta?

**FIGURE 2:**
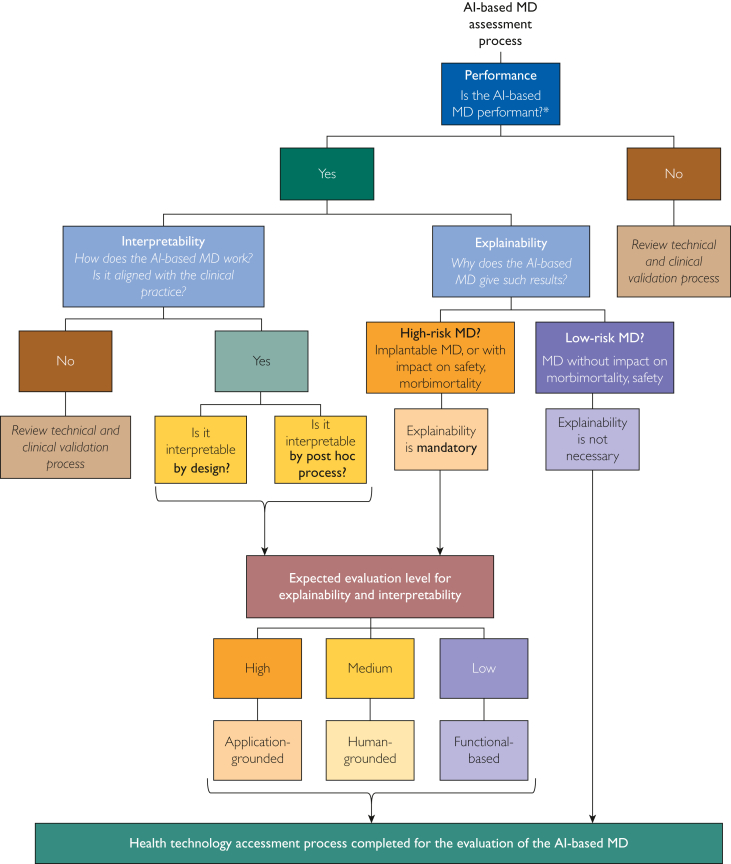
Flowchart summarizing the recommendations relative to the type of assessment tool that could be used depending on each criterion analyzed in our article, that is, performance, interpretability, and explainability for technology assessment of artificial intelligence (AI)-based medical devices (MDs). ∗Assuming the concomitant evaluation of other health technology assessment criteria, such as safety.

To sum up the HTA process of AI-based MDs, we established a flowchart that maps our recommendations toward the type of assessment tool that could be used depending on each criterion analyzed in the present article (Figure 2). We assumed that concomitant evaluation of other HTA criteria, such as safety, would be undertaken at the same time. Performance and interpretability should be evaluated for each category of AI-based MDs, whereas explainability might not be mandatory for low-risk AI-based MDs (in contrast to high-risk MDs), that is, devices with no impact on morbimortality or safety.

### Complex Trade-off Between Performance and Interpretability and Explainability

The predictive performance of AI systems is a key issue. However, the importance of explainability depends on the specific AI and its intended use. If explainability is not important and if a black-box model could be acceptable, the model with the best predictive performance is more interesting because explanations can be expensive. When a model has a high level of explainability, the selection of explainable AI methods could be considered.^8^^,^^9^^,^^57^^,^^58^ It is difficult to satisfy all properties of explainability. Holzinger et al^59^ suggested a brief overview of 17 explainable AI methods, including Local Interpretable Model-agnostic Explanations, Anchors, Graph Local Interpretable Model Explanations, Shapley Flow, Textual Explanations of Visual Models, Integrated Gradients, Causal Models, or Meaningful Perturbations. For instance, Arras et al^60^ proposed to adapt the Layer-wise Relevance Propagation technique used for explaining the predictions of feed-forward networks to the Long Short Term Memory architecture used for sequential data modeling in healthcare. Thus, as the developer of an AI system, it is important to establish the relative importance of explainability compared with predictive performance and what is desired by end users of the AI system.

### Performance, Interpretability, and Explainability: Key Requirements for a Trustworthy AI

At an international level, healthcare professionals seem to have difficulties trusting AI-based MDs. A study by Oh et al^61^ highlighted that only 5.9% of Korean doctors reported having good familiarity with AI. Among 999 Japanese physicians interviewed, only 44.7% expressed an intention to use AI-driven medicine.^62^ Another study showed that companies require more data, funding, and regulatory certainty, and clinicians and patients insist on trustworthy AI-based MDs.^63^

There are several issues that can decrease physicians’ trust in AI in their clinical practice, such as the low number of randomized clinical trials assessing the performance of AI-based MDs, the lack of transparency within these technologies, the risk of inequity introduced by AI biases, and insufficient regulatory clarity.^12^ The need for trustworthy AI exponentially increased in the healthcare ecosystem with the several considerations in medical imaging, as Hasani et al^11^ highlighted with a proposition of 14 core principles to promote trustworthy AI-based MDs in medical imaging, such as transparency, explainability, technical robustness, or stakeholders involvement. Holzinger et al^64^ insisted on bridging the gap between research and practical applications in the context of future trustworthy medical AI with human-centered AI design methods.

According to Ossa et al,^47^ explainability needs to be sufficient but not exhaustive for doctors and patients. The acceptable standards for explainability are context-dependent and rely on the risks of the clinical scenario, and factors that form part of AI’s explainability include usefulness and uncertainty, risk of bias, responsibility attribution, and the AI’s involvement in decision making.

To provide interpretability, methodologies for explainable AI need to be associated with ethical and legal analysis.^65^, ^66^, ^67^, ^68^, ^69^ For instance, Currie et al^70^ confirmed the need of addressing the ethical and legal challenge of AI in nuclear medicine. Naik et al^71^ showed that as we rely more on AI for decision making, it becomes important to ensure that they are made ethically and free from unjust biases to tackle the responsible AI notion with devices that are transparent, explainable, and accountable.

### A Regulatory Need Toward Responsible AI

The 3 notions that we covered in this article are also part of the process of creating confidence in AI in healthcare. The level of confidence in an algorithm in fact relies heavily on transparency (interpretability and explainability of outputs) and ethics (in terms of trustworthiness and regulation).^72^

The work by Liao et al^26^ led to the identification of diverse motivations based on AI users’ needs, such as gaining further insights for decision making, appropriately evaluating algorithm capability, and highlighting the ethical responsibilities of AI products. The lack of explanation for some “black-box” algorithms raises ethical questions, particularly in healthcare.^27^ Closely related concepts are fairness and ethical AI. Fairness refers to the idea that an algorithm should make predictions that are unbiased and do not discriminate against any group of individuals.^73^ Ethical AI describes the use and design of an algorithm that are in line with human values and the rights and well-being of individuals.^65^ The relationship between such concepts is that interpretability and explainability can help to strive toward fairness and ethical AI. Providing interpretability and explainability for an algorithm’s predictions typically means bringing forward transparency and accountability by detecting (and addressing) potential biases or ethical issues (even though some explanations can hide unfairness, as underlined by Dimanov et al^74^ and Slack et al^75^). In this way, stakeholders can better understand how the algorithm works and can evaluate whether fair and unbiased decisions are made.^76^ The aim of transparency and explainability of AI-based MDs hence contributes to fair and accountable algorithmic decision-making processes.^77^

For these reasons, initiatives are awaited from institutions. For instance, the Confiance.ai program was launched in July 2021 and gathers 13 private and public institutes. Together, they aim to build a trusted AI in the industry to ensure the reliability, security, and certification of AI-based systems.

## Conclusion

After the identification of 3 main assessment criteria for AI-based MDs according to HTA guidelines, we provided a set of tools and methods to help understand how and why ML algorithms work as well as their predictions. We also highlighted the increase in the importance of explainability and interpretability techniques for HTA agencies to hold stakeholders more accountable for the decisions made by AI-based MDs given how crucial such understanding is in high-stakes decisions. Finally, we believe that raising awareness of these concepts is essential for their widespread adoption and confidence.

## Potential Competing Interests

Author Murris reports a grant from the 10.13039/501100003032Association Nationale de la Recherche et de la Technologie, with 10.13039/100013226Pierre Fabre, Convention industrielle de formation par la recherche number 2020/1701.
