# Supplementary material for: Assessment of Performance, Interpretability, and Explainability in Artificial Intelligence–Based Health Technologies: What Healthcare Stakeholders Need to Know
Source: Mayo Clin Proc Digit Health. 2023 Apr 21;1(2):120–38. doi: 10.1016/j.mcpdig.2023.02.004 (PMC11975643; doi:10.1016/j.mcpdig.2023.02.004)
Supplement: Supplementary Materials [file mmc1.pdf]

Supplementary Material

1 Literature review protocol

- **Search strategy and study identification**

Comprehensive research was done following the Preferred Reporting Items for Systematic Reviews and Meta-Analyses (PRISMA) methodology with the following search terms: "health technology assessment"[All Fields] AND ("artificial intelligence"[All Fields] OR "machine learning"[All Fields] OR "deep learning"[All Fields])).[1] We searched the PubMed databases and HTA agency websites. Research was limited to English and French language publications.

- **Inclusion and exclusion criteria**

Original research articles, guidelines, and HTA recommendations assessing AI-based MDs were included. Editorials, letters, comments, newspaper articles, and posters were excluded.

- **Article selection**

The selection was conducted by two reviewers (LF & JM). After removing duplicates, they independently screened abstracts to select potentially eligible studies. Full texts were then analyzed for eligibility. A third reviewer (NM) resolved any discrepancies highlighted during the selection process, if consensus was not reached. An extraction database was used to list the selected studies meeting the inclusion criteria.

- **Data extraction**

The following items were extracted from the selected articles:

- General characteristics of the study (authors, country, publication date, journal)
- Characteristics of the AI technology
- Criteria for HTA
- Description of criteria

[1] Page, M. J. *et al.* The PRISMA 2020 statement: an updated guideline for reporting systematic reviews. *BMJ* **372**, n71 (2021).

2 Hyperparameter optimization

Settings of the learner are commonly called hyperparameters and help in controlling the learning process.[2] Hyperparameter optimization is the process of finding the hyperparameter configuration that produces the best performance. The idea is to find the hyperparameter combinations that maximize the CV metric. Various approaches include the following (non-exhaustive list):

- GridSearch CV exhaustively considers all parameter combinations from a given hyperparameter space of values;[3]
- RandomSearch CV samples a number of trials from a given hyperparameter space of distributions;[4]
- Bayesian CV is similar to GridSearch CV and considers Bayesian optimization.[5]

All CV methodology may also be used in nested or halving search frameworks, and softwares like Optuna were specifically designed to enhance the optimization process.[6,7,8] There is no consensus to date on which optimization approach to use; the choice relies on the data and computation power.[9,10] The idea is to find a compromise between computation time and the level of optimization expected; everything is about equilibrium between the two. Take-home messages also include that bias may come from the choice of the model family and not only from a specific hyperparameter and that high variance is not related to a specific structure for the prediction error.[11,12]

---

[2] Probst, P., Boulesteix, A.-L. & Bischl, B. Tunability: importance of hyperparameters of machine learning algorithms. *J. Mach. Learn. Res.* **20**, 1934–1965 (2019)
[3] Lerman, P. M. Fitting Segmented Regression Models by Grid Search. *Journal of the Royal Statistical Society. Series C (Applied Statistics)* **29**, 77–84 (1980).
[4] Bergstra, J., & Bengio, Y. (2012). Random search for hyper-parameter optimization. Journal of machine learning research, 13(2).
[5] Watanabe, S., & Opper, M. (2010). Asymptotic equivalence of Bayes cross validation and widely applicable information criterion in singular learning theory. Journal of machine learning research, 11(12).
[6] Jamieson, K., & Talwalkar, A. (2016, May). Non-stochastic best arm identification and hyperparameter optimization. In Artificial intelligence and statistics (pp. 240-248). PMLR.
[7] Cawley, G. C., & Talbot, N. L. (2010). On over-fitting in model selection and subsequent selection bias in performance evaluation. The Journal of Machine Learning Research, 11, 2079-2107.
[8] Akiba, T., Sano, S., Yanase, T., Ohta, T., & Koyama, M. (2019, July). Optuna: A next-generation hyperparameter optimization framework. In Proceedings of the 25th ACM SIGKDD international conference on knowledge discovery & data mining (pp. 2623-2631).
[9] Yu, T. & Zhu, H. Hyper-Parameter Optimization: A Review of Algorithms and Applications. *ArXiv* (2020).
[10] Bischl, B., Binder, M., Lang, *et al.* (2021). Hyperparameter optimization: Foundations, algorithms, best practices and open challenges. arXiv preprint arXiv:2107.05847..
[11] Belkin, M., Hsu, D., Ma, S. & Mandal, S. Reconciling modern machine learning practice and the bias-variance trade-off. *Proc. Natl. Acad. Sci. U.S.A.* **116**, 15849–15854 (2019).
[12] Feurer, M., & Hutter, F. (2019). Hyperparameter optimization. In Automated machine learning (pp. 3-33). Springer, Cham.
