# Supplementary material for: Assessment of Performance, Interpretability, and Explainability in Artificial Intelligence–Based Health Technologies: What Healthcare Stakeholders Need to Know
Source: Mayo Clin Proc Digit Health. 2023 Apr 21;1(2):120–38. doi: 10.1016/j.mcpdig.2023.02.004 (PMC11975643; doi:10.1016/j.mcpdig.2023.02.004)
Supplement: Supplementary Table [file mmc2.pdf]

Supplementary Table A Evaluation metrics based on outcome

Legend – AIC = Akaike information criterion, AUC = Area under the receiver operating characteristic curve, BIC = Bayesian information criterion, C-index = Concordance index, MAE = Mean Absolute Error, RMSE = Root-mean-square error; RMSLE = Root-mean-square log error

Continuous outcome	Categorical outcome	Time-to-event outcome
AIC / BIC*<br>MAE<br>RMSE / RMSLE<br>R2 / Q2	Brier score<br>R2-like measures<br>Derivatives from the confusion matrix: accuracy, precision, recall, F1 score, specificity, sensitivity<br>AUROC	RMSE<br>Integrated Brier score<br>D-calibration<br>C-indices: Harrell’s C-index, Uno’s C-index, Kim’s C-index

*AIC and BIC shall be used to measure goodness of fit only.
