# Supplementary material for: Assessment of Performance, Interpretability, and Explainability in Artificial Intelligence–Based Health Technologies: What Healthcare Stakeholders Need to Know
Source: Mayo Clin Proc Digit Health. 2023 Apr 21;1(2):120–38. doi: 10.1016/j.mcpdig.2023.02.004 (PMC11975643; doi:10.1016/j.mcpdig.2023.02.004)

## Artificial intelligence ecosystem mapping in healthcare in Europe

The AI ecosystem involves several stakeholders with heterogeneous capabilities to develop, validate, assess, deploy and use AI-based MDs with new stakeholders (in data, information technology, and engineering: AI public research institutes).

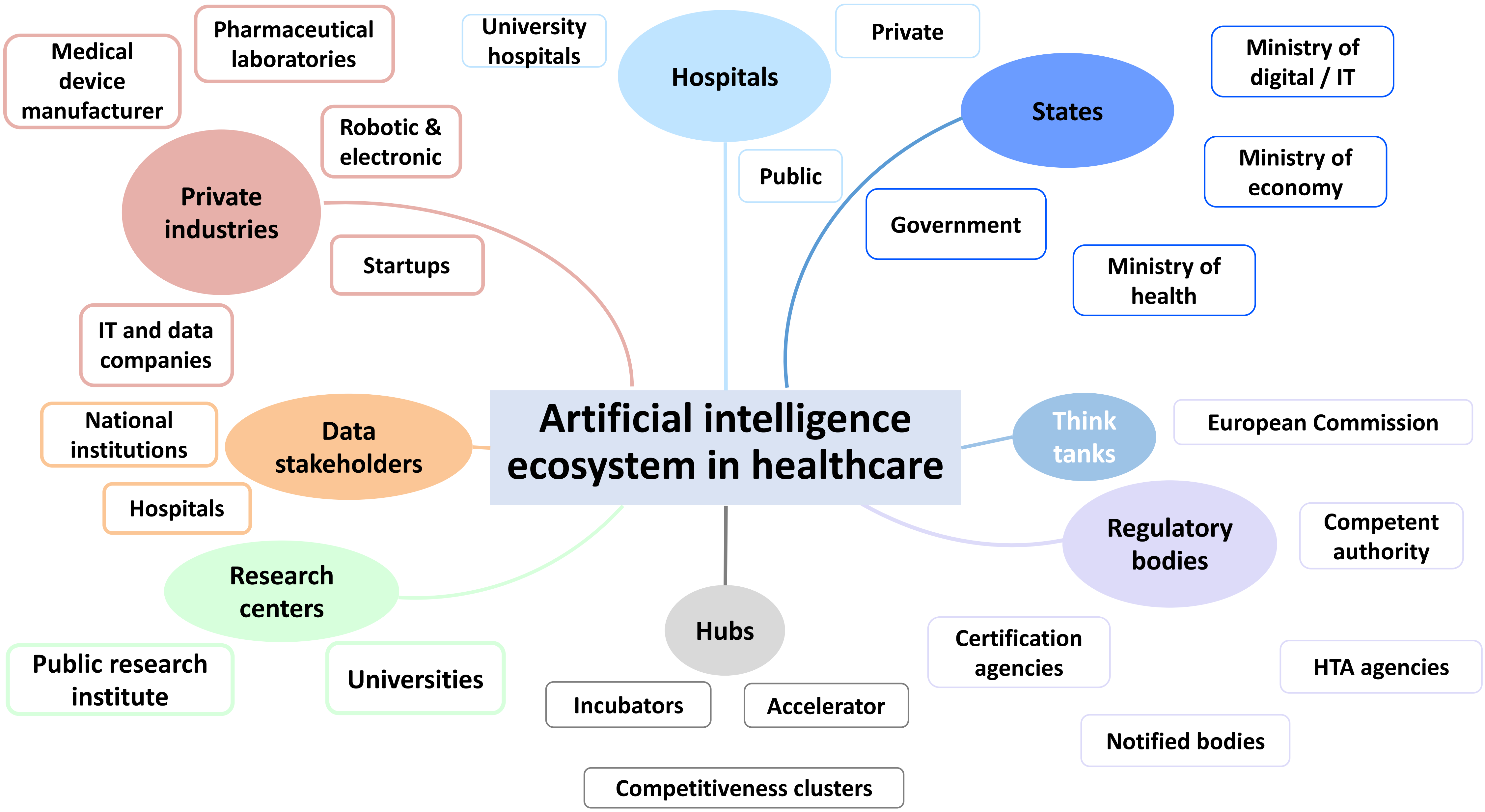

Supplement: Supplementary Figure 1 [file mmc3.pdf]
