# Supplementary figures and images for: Assessment of Performance, Interpretability, and Explainability in Artificial Intelligence–Based Health Technologies: What Healthcare Stakeholders Need to Know
Source: Mayo Clin Proc Digit Health. 2023 Apr 21;1(2):120–38. doi: 10.1016/j.mcpdig.2023.02.004 (PMC11975643; doi:10.1016/j.mcpdig.2023.02.004)

## Use of data in the performance evaluation process for AI/ML algorithms

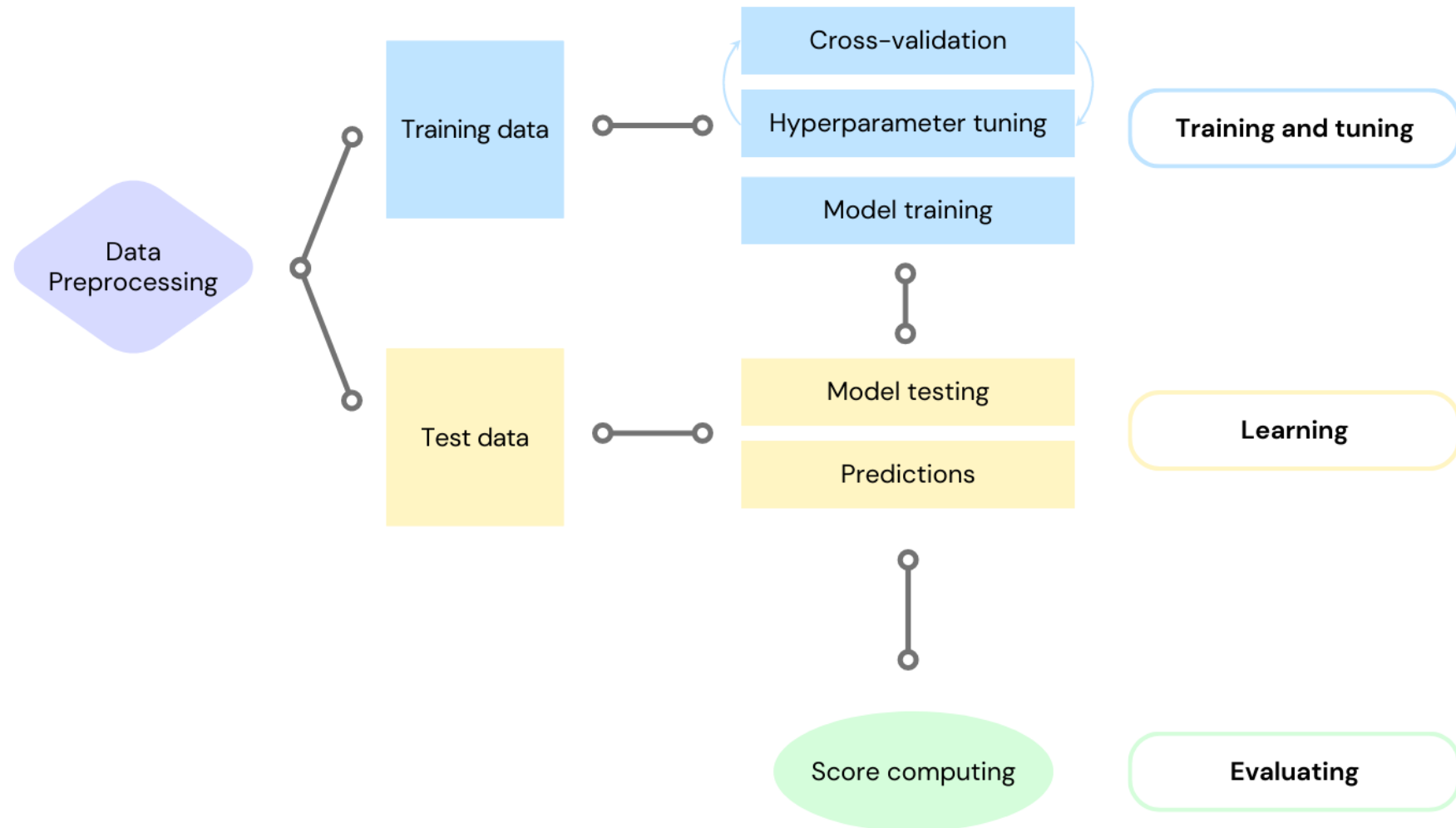

Supplement: Supplementary Figure 2 [file mmc4.pdf]
